# The Metric System in Medicine

**Published:** 1878-09

**Authors:** Edw. Wigglelworth

**Affiliations:** Boston; Instructor in Harvard Medical School and Physician to Boston City Hospital


ATLANTA
Medical and Surgical Journal.

VOL. XVI.] SEPTEMBER—1878. [No. 6

### Original Communications.

### THE METRIC SYSTEM IN MEDICINE.

BY EDW. WIGGLELWORTH, A. M., M. D.
Instructor in Harvard Medical School and Physician to Boston City Hospital.

*Read before the Amer. Med. Association [Section of Practical Medicine, Materia Medica and Physiology] at Buffalo, June 5th*, 1878.

In answer to the common question "of what special advantage will the metric system be to me, and what ought I to do to advance its introduction," it may be said that every one should do all in his power to further its introduction, since it possesses such great merits in general; that the best way to accomplish this is for each branch of science or education, every profession, trade, and occupation to promote the interests of the system to the extent of its power within its own sphere, in order that the sum of all these social parts may equal the whole community; and to be able to give a reason for the faith which is in it by the testimony, based on experience, of its individual members or adherents.

The special advantage accruing to one such member of a profession accrues to all its members, and the reason why the system is so rapidly gaining ground in the medical profession is well given in the Metric Bulletin for November, 1876:

I. "Because of its great convenience in writing and compounding prescriptions, in dividing doses, and in computing quantities required during given times.

II. "Because physicians have learned what the system really is in their chemical studies, and to thoroughly understand it is to wish to adopt it.

III. "Because from the cosmopolitan character of medicine, a prescription from any one of the score of countries using the system is liable to find its way into a physician's note book, and it is a great convenience to be able to send it to the druggist without spending an hour in adopting it to our tangle of measures.

IV. "Because our medical men more than almost any other class, are free from that jealous conservatism and bigotry which forbid a fair examination of a thing, though it promises the greatest good, simply because it was not in the creed of their fathers.

"Weights and measures enter so little into the everyday life of the other learned professions—law and theology—that the interest in those quarters must be prompted by pure philanthropy, while every physician has a practical personal interest in the adoption.

"The druggists who have facilities for weighing and measuring in the metric system report a constantly increasing number of prescriptions written in the new form; and we may look to be cured (or killed) in accordance with the international measures before our clothes are made or our provisions dispensed in its denominations."

Scientific medicine has long employed the metric system as a matter of course; fine work demanding the best of tools. That the domestic drudgery of the general practitioner—prescription writing—stands also in need of the system is well shown by documents presented to the Comitia Minora of the county society of New York, which at the annual meeting, November 27, 1875, reported to the society the following resolution, which was adopted:

*Resolved*, That the Medical Society of the county of New York recommends to its members the use of the metric system in their prescriptions.

And also by subsequent editorials in the New York Medical Record based on these documents (New York Med. Record, Dec. 9, 16, 23, 30, 1876.) Thus, in the old method of prescribing, various units are employed, viz.:

the grain, scruple, drachm, and ounce, as measures of weight, and the fluidrachm, fluidounce, and pint, as measures of capacity. These measures of capacity bear the same name in England, but represent very different amounts; a source of great inconvenience in reading English books, and a source of practical danger even, since English made graduates are said to be imported and employed by American pharmacists.

There is another difficulty arising from the use of the same terms for both measure and capacity. One *fluid* ounce weighs 435.6 grains. Should the physician omit to place an *f* before the ounce-symbol (and many never insert this prefix at all,) it is the druggist's duty to supply a Troy ounce or 480 grains of the preparation demanded. The German druggists, as the rule, do this; the independent American takes it for granted that fluidounce is meant, and supplies that. The English solid ounce again is different style, being the avoirdupois ounce of 437.5 grains. The strength of ostensibly similar solutions of powerful drugs may thus be made to vary greatly.

Then, too, accurate measurements of capacity by means of graduated glasses are very difficult at best, while the ease in employing them leads to great carelessness, which would be obviated were weights alone employed. Moreover, the graduating glass themselves are roughly made, and sold at three dollars a dozen, showing that very little time or trouble has been spent upon their correct adjustment. A large proportion of the Troy weights in use in this country are also inaccurate, many of them seriously so; inspectors of weights not examining the prescription weights, but merely the avoirdupois. The same is true of the balances employed, many being incorrect; some not turning with fine weights, which are consequently estimated, while in other cases weights above half an ounce have been estimated by avoirdupois instead of Troy weight.

"The practice of medicine necessarily presents so many uncertainties that we should make an effort, if possible, to diminish these; and if we can secure uniformity in the dispensing of our medicines, we can eliminate at least one variable factor.

"In the metric system we deal with but one unit—the gram—equal to a little less than fifteen and a half grains Troy. All integral quantities are expressed in multiples of this, and all fractions in decimals, the decimal-point being always placed in its proper position between the integers and fraction, in the same way that we place the point between dollars and cents in our own monetary notations; and this is the whole of the metric system so far as it immediately concerns us. It is expedient for the present to write the word grams in full over the first line of figures." Instead of the decimal-point a perpendicular *line*, as suggested by Dr. W. Bolles, should be drawn from the top to the bottom of the right side of the prescription paper.

Drops might be allowable also in metric prescriptions, since we are to lose the minim, for which, practically, though not with justice, the drop is usually substituted.

Another merit of the metric system is its convenience in calculating and altering formulas (metric) when it is desirable to increase or diminish the quantity of the active ingredients; the quantity of the vehicle and of the dose remaining as at first.

The majority of objections to the new system have been raised by ignorant people, and are based upon misapprehension; thus,

I. "The system is complicated and difficut to learn." On the contrary, its simplicity is a great feature, and it can be fully acquired in half an hour.

II. "It will throw us out of uniformity with English practice." Only, however, as to weight. Our measures are different at present, and it throws us *into* uniformity with nearly all other countries; namely, with populations aggregating four hundred and thirty-two millions, whereas the population of England is only about thirty-two millions. By other populations numbering forty-eight millions it is already partially employed. Moreover, our best medical literature comes not from England, but from those countries—Germany especially—which use the metric system.

III. "We must forget the units of length, volume, and weight to which we have been accustomed." Not at all; we merely *acquire new ones*, and employ these, and we shall very soon insensibly begin to think in the new system; which is to be acquired in the same way as a new language—namely, by beginning with it as we began with our own.

IV. "The druggists are not familiar with the metric system." On the contrary, they are far in advance of the physicians, and strongly in favor of its adoption.

V. "The druggists have not the requisite weights, and will calculate back into the old system and may make mistakes." All the best druggists have metric weights already. Those not having them can obtain complete sets for about six dollars—a sum which a single day will restore to them from their gain in metric prescriptions. We may also feel sure, as suggested by Dr. A. N. Blodgett (*Boston Medical and Surgical Journal*, December 21, 1876,) of "better drugs from a pharmacist educated to use this system than from one who has acquired his experience as a trade learned from his master. The metric system, therefore, seems to offer the following distinct and definite advantages: It dispenses with the signs of the quantities; it employs Arabic figures instead of Roman numerals; it assures the physician of more competent service from the pharmacist, and of a better quality of medicine, and reduces considerably the danger of mistakes on the part of the physician and druggist."

"It provides denominations of weights applicable to the smallest quantity which the physician or the pharmacist can be called upon to prescribe or dispense, the old grain being by far too large a unit for the measurement of the powerful drugs which modern chemistry has added to our materia medica. It is decimal in the character of its divisions, and we all recognize the facility of decimal arithmetical operations. By this arithmetical simplicity we are enabled more readily to appreciate quantitive ratios in our formulas, and in the resulting pharmaceutical preparations. It enables us to substitute magistral for officinal recipes, and if these were more extensively used, comparatively simple formulas would be readily improvised to meet the varying exigencies of individual cases, the tendency to polypharmacy would be diminished, the study and practice of rational therapeutics would be facilitated, and the extensive use of patent medicines by physicians, which is the opprobium of the medical profession in this country, would be lessened."—*T. B. Curtis, in the Boston Medical and Surgical Journal, Dec.* 6, 1877.

The metric system alone furnishes sufficient accuracy for the chemist and pharmacist. It is necessary in the microscopic measurements of the anatomist and physiologist, and in the microscopic ones of the surgeon. It gives to general therapeutics the centigrade thermometer and other instruments, and becomes daily more needful as general medicine crystallizes into specialties with their naturally and necessarily greater acquirements in science, and consequently greater requirements from art.

Thus, for example, Dr. E. G. Loring (Boston *Med. and Surg. Journal*, Sept. 7, 1876) and Dr. H. Derby (Boston *Med. and Surg. Journal*, Oct. 12, 1876), allude to the great superiority of the metric system in ophthalmology. Dr. Derby says, for instance, among various reasons for adopting the metric system, that the numeration of spectacle lenses is now based upon the metric system, and that the loss of one's eye-glasses in any part of the civilized world involves merely the expense of a new pair with the focal distance and other attributes of the lost lenses. Formerly the unit of the system in use was a one-inch lens, which was too strong for practical purposes, and therefore had no real existence. Moreover, this very system was variable, and to ascertain the strength of a lens of a given number, especially of a high one, it was necessary to find out in what country it was ground. The Paris inch differed from the English 36.94 of the former, 36.37 of the latter going to an inch. Between these came the Rhenish and Austrian inches, all different from each other. A patient asking for No. 2 in London, Paris, Berlin or Vienna, might receive a different glass in each place.

At a meeting of the Heidelberg Society in 1875, and shortly afterward at the Medical Congress at Brussels, Prof. Donders proposed the metric as the system of numeration. It has been adopted by the most eminent ophthalmologists, and its use is daily spreading. In our own country the surgeons of the Boston Eye and Ear Infirmary voted in 1876, "that all future measures of length and refraction be recorded in and all glasses ordered on the metric system."

The advantage of this uniform numeration of spectacle lenses is obvious. Whereas, in old times, a No. 2 lens meant a lens of two (English, French, Austrian or Prussian) inches focal length, according to the country in which it was ground, it now means a lens equal to two lenses, and having a focal length of one meter, *i. e.*, a lens of fifty centimetres focal length.

Finally, in regard to volumetric or gravimetric methods, supposing the metric system to be adopted, it is a purely subsidiary question how we shall use it—that is, whether by weight or measure in prescribing. Measures may be used just as we use them at present in our "systemless system." But it is better to make the complete change at once, since we must come eventually to prescribing by weight alone; for

1. Prescribing by weight gives more exact results.
2. When once learned it is vastly more convenient.
3. It is perfectly easy to learn.

The only difficulty is in the apportionment of doses, since we must continue to allow the patient to take his medicine by the domestic measurement of spoonfuls. Now, these doses once learned will be more easily remembered by weight than by volume, since they will have been made in fixed proportions by weight only, and in the same way prescriptions as a whole will be more readily borne in mind. But there is no need even of this, for there is no trouble in estimating the volume, and consequently the dose, of fluids prescribed by weight; for whether reached by the use of weights or measures, the result is in either case the same definite bulk of menstruum, and is to be divided up, of course, in the same manner for doses. Thus the bulk of a mixture is either an infusion, a tincture, water, or a syrup. Of these the first three have specific gravities, which are, for practical purposes, identical.

The fourth is a third heavier than the others. What is, then, simpler than, when prescribing by weight, to prescribe of a syrup four thirds as much by weight as if we were prescribing plain water, which bulk can then be divided up by the patient into his time-honored "teaspoonful three times a day." Other liquids, the specific gravities of which vary markedly from that of water, are very simply never prescribed in bulk, and therefore need not be considered. The increased accuracy derived from the employment of weights more than compensates for any slight variation in the small amount of these liquids in a single dose of menstruum—a variation less than that in the idiosyncracies of different patients, or would even justify the plan proposed by Mr. Alfred Taylor, of "making the mixture up to a desired bulk by adding sufficient quantity of the vehicle or adjuvant." Moreover, fixed oils, liquids, acids and chloroform are by our present pharmacopœia directed to be always weighed. Why, then, stop these, since we need equal accuracy in regard to other fluids?

If the physician prefers, however, to be strictly accurate on principle, this also is possible. Prof. Maisch has called attention to the fact that neither the drop, the teaspoon nor the tablespoon possesses the exact value in bulk usually assigned to it; that evaporation and adhesion militate against accuracy in the use of volumetric methods, it even being difficult on account of the refraction of light and the thickness and coarseness of the measuring glass, to correctly ascertain the height of the liquid in such glass; that consequently gravimetric methods are preferable; and in the *American Journal of Pharmacy* for February 1, 1877, he discusses these points at length, and "shows that the physician may gradually accustom himself to the changes, either by using the table there given in converting the measures of different liquids into their corresponding metric weights, or by directing the apothecary to dispense them by this weight after converting the weight and measure now in use into grains."—*Boston Med. and Surg. Journal, April* 5, 1877.

To conclude, the gravimetric method is the one employed by all nations using the metric system, and it is of the highest importance to avoïd courting a disagreeable notoriety by an affected and purposeless singularity, based upon indolence and selfishness. As the church with its common tongue, the Latin, is at home wherever churchism extends, so science, by promoting universally identical customs, thoughts and language, must hasten the approach of universal brotherhood, peace, knowledge, and consequently happiness.

After this paper had been read, the following resolution was unanimously adopted:

*Resolved*, That this section, recognizing the value of the metric system for its uniform, international, indestructible, generally applicable, convenient, simple, safe and scientific character, hereby recommend to all physicians the use of the same in their practice, and in their writings and teachings.

